# Non-singular space-times with a negative cosmological constant: II. Static solutions of the Einstein–Maxwell equations

**DOI:** 10.1007/s11005-017-0955-x

**Published:** 2017-03-14

**Authors:** Piotr T. Chruściel, Erwann Delay

**Affiliations:** 1 Faculty of Physics and Erwin Schrödinger Institute, University of Vienna, Boltzmanngasse 5, 1090 Vienna, Austria; 2 Laboratoire de Mathématiques d’Avignon (EA 2151), Avignon Université, 84018 Avignon, France

**Keywords:** Stationary metrics, Einstein–Maxwell equations, Negative cosmological constant, 83C20, 83E15

## Abstract

We construct infinite-dimensional families of non-singular static space-times, solutions of the vacuum Einstein–Maxwell equations with a negative cosmological constant. The families include an infinite-dimensional family of solutions with the usual AdS conformal structure at conformal infinity.

## Introduction

Stationary and static solutions play a fundamental role in any theory. Stable such solutions provide families of possible end states of the problem at hand. Unstable ones display features of the solutions that are likely not to be encountered at late times of evolution.

Lichnerowicz’s theorem asserts that the only vacuum stationary well-behaved solution with $\Lambda =0$ is Minkowski space-time. It therefore came as a surprise that there exist many stationary solutions of the vacuum Einstein equations with negative cosmological constant which have a smooth conformal structure [3, 4, 8]. In particular, there exist many globally well-behaved stationary vacuum space-times with negative cosmological constant which have no symmetries whatsoever.

Now, Lichnerowicz’s theorem generalises to static Einstein–Maxwell equations with $\Lambda =0$ [14]: there is again only one such regular solution, namely Minkowski space-time. It is likewise of interest to enquire what happens when $\Lambda <0$. In this work, we show that, similarly, no Lichnerowicz-type theorem exists in this case: we prove existence of infinite-dimensional families of static metrics satisfying the Einstein–Maxwell equations with a negative cosmological constant and admitting a smooth conformal completion at infinity.

More precisely, we show that a large class of such fields can be constructed by prescribing the conformal class of a static Lorentzian metric and the asymptotic behaviour of the electric field on the conformal boundary $\partial \mathscr {M}$, provided that the boundary class is sufficiently close to, e.g. that of anti-de Sitter space-time and the freely prescribable leading coefficient in an asymptotic expansion of the electric potential is sufficiently small. This complements our previous work on vacuum metrics in [8], which we think of as paper I in this series.

The key new feature of the current result, as compared to [3, 4, 8], is that we can drive the solution with the electric field maintaining, if desired, a conformally flat structure at the conformal boundary at infinity.

Once this work was completed we have noticed [7], where a class of numerical solutions of the Einstein–Maxwell equations with $\Lambda <0$ is presented. Our work justifies rigorously the existence of weak-matter-fields configurations within the family constructed numerically in [7] and provides many other static non-vacuum solutions near anti-de Sitter.

We thus seek to construct Lorentzian metrics ${}{{\mathbf {g}}}$, solutions of Einstein–Maxwell equations with a negative cosmological constant $\Lambda$, in any space-dimension $n\ge 3$. More precisely, we consider the following field equations for a metric

$$\begin{aligned} {{\mathbf {g}}}=g_{\mu \nu }\mathrm{d}x^\mu \mathrm{d}x^ \nu \end{aligned}$$

and a two-form field *F* in space-time dimension $n\,+\,1$, $n\ge 3$ generalising the Einstein–Maxwell equations in dimension $3+1$ as

1.1 $$\begin{aligned} {\text {Ric}}({{\mathbf {g}}})-\frac{ {\text {Tr}}{\text {Ric}}({{\mathbf {g}}})}{2} {}{{\mathbf {g}}}+\Lambda {}{{\mathbf {g}}}=F\circ F-\frac{1}{4}\langle F,F\rangle _{{{\mathbf {g}}}}{{\mathbf {g}}}, \end{aligned}$$

where ${\text {Tr}}$ denotes a trace with the relevant metric (which should be obvious from the context), $(F\circ F)_{\alpha \beta } := g^{\mu \nu } F_{\alpha \mu } F_{\beta \nu }$, and

$$\begin{aligned} \langle F,F\rangle \equiv g^{\alpha \beta } g^{\mu \nu } F_{\alpha \mu } F_{\beta \nu }=: |F|^2. \end{aligned}$$

This is complemented with the Maxwell equations

1.2 $$\begin{aligned} \text{ div }_{{{\mathbf {g}}}}F=0 = \mathrm{d}F. \end{aligned}$$

We further assume existence of a hypersurface-orthogonal globally timelike Killing vector $X=\partial /\partial t$. In adapted coordinates, the space-time metric can be written as

1.3 $$\begin{aligned} {{\mathbf {g}}}= & {} -V^2\mathrm{d}t^2 + \underbrace{g_{ij}\mathrm{d}x^i \mathrm{d}x^j}_{=g}, \end{aligned}$$

1.4 $$\begin{aligned} \partial _t V= & {} \partial _t g=0. \end{aligned}$$

We further assume that the Maxwell field takes the form

1.5 $$\begin{aligned} F=\mathrm{d}(U\mathrm{d}t), \quad \partial _t U =0. \end{aligned}$$

Our main result reads as follows (see Sect. 2 below for the definition of non-degeneracy; the function $\rho$ in (1.7) is a coordinate near the conformal boundary at infinity $\partial M$ that vanishes at $\partial M$):

### Theorem 1.1

Let $n=\dim M\ge 3$, $k\in {\mathbb {N}}\smallsetminus \{0\}$, $\alpha \in (0,1)$, and consider an Einstein metric $\mathring{{{\mathbf {g}}}}$ as in (1.3)–(1.4) with strictly positive $V={\mathring{V}}$, $g={\mathring{g}}$, such that the associated Riemannian metric $\mathring{{{\mathfrak {g}}}}={{\mathring{V}}}^2\mathrm{d}\varphi ^2+{\mathring{g}}$ on $\mathbb {S}^1\times M$ is $C^2$-compactifiable and non-degenerate, with smooth conformal infinity. For every smooth $\widehat{U}$, sufficiently close to zero in $C^{k+2,\alpha }(\partial M)$, there exists a unique, modulo diffeomorphisms which are the identity at the boundary, solution of the static Einstein–Maxwell equations of the form (1.3)–(1.5) such that, in local coordinates near the conformal boundary $\partial M$,

1.6 $$\begin{aligned} V-{\mathring{V}}= & {} O(\rho ),\quad g_{ij}-{\mathring{g}}_{ij} =O(1), \end{aligned}$$

1.7 $$\begin{aligned} U= & {} {\widehat{U}} +\left\{ \begin{array}{ll} O(\rho ), &{} n=3; \\ O(\rho ^2 \ln \rho ), &{} n=4;\\ O(\rho ^2), &{} n\ge 5 . \end{array}\right. \end{aligned}$$

### Remark 1.2

We have emphasised the freedom to choose the leading-order behaviour ${\widehat{U}}$ of *U*. As such, the leading-order behaviour of both ${\mathring{V}}$ and ${\mathring{g}}$ is, similarly, freely prescribable near a non-degenerate solution [4, 8]. Here, one can, if desired, proceed in two steps: first, find the static solution with new fields ${\mathring{V}}$ and ${\mathring{g}}$ near a non-degenerate solution; small such perturbations will preserve non-degeneracy. One can then use Theorem 1.1 at the perturbed static solution to obtain a solution with the desired small ${\widehat{U}}$. $\square$

The $(n+1)$-dimensional anti-de Sitter metric is non-degenerate in the sense above (see eg. [4], Appendix D), so Theorem 1.1 provides in particular an infinite-dimensional family of solutions near that metric. We note existence of further large classes of Einstein metrics satisfying the non-degeneracy condition [1, 2, 4, 15].

The requirement of strict positivity of ${\mathring{V}}$ excludes black hole solutions, and we are hoping to return to the construction of black-hole solutions in a near future. In fact, this work was prompted by [13], where static Einstein–Maxwell black holes solutions driven by the asymptotics of the electric field have been constructed numerically.

Similarly to [13], for generic $\widehat{U}$ the resulting space-time metric will have no isometries other than time-translations.

When the free boundary data ${\widehat{U}}$ are smooth and when ${\mathring{{{\mathfrak {g}}}}}$ is conformally smooth, the solutions constructed here will have a polyhomogeneous expansion at the conformal boundary at infinity. The proof of this is an immediate repetition of the argument presented in [8, Section 7], where the reader can also find the definition of polyhomogeneity. The decay rates in (1.6)–(1.7) have to be compared with the local-coordinates leading-order behaviour $\rho ^{-2}$ both for ${{\mathring{V}}}^2$ and ${{\mathring{g}}}_{ij}$. A more precise version of (1.6)–(1.7) in terms of weighted function spaces (we follow the notation in [8, 15]) reads, in all dimensions,

1.8 $$\begin{aligned}&\displaystyle (V-{\mathring{V}})\in C_{1}^{k+2,\alpha }( M),\,(g-{\mathring{g}})\in C_{2}^{k+2,\alpha }(M,{{\mathcal {S}}}_2), \end{aligned}$$

1.9 $$\begin{aligned}&\displaystyle U-{\widehat{U}} \in C_{1}^{k+2,\alpha }( M), \end{aligned}$$

and the norms of the differences above are small in those spaces. To this, one can add, in dimensions $n\ge 4$,

1.10 $$\begin{aligned} U-{\widehat{U}} - U_{\ln }\, \rho ^2 \ln \rho \in C_{2}^{k+2,\alpha }( M) , \end{aligned}$$

with the function $U_{\ln } \in C^\infty ( \mathbb {S}^1\times \overline{M})$ given by (5.4) below when $n=4$, and $U_{\ln } \equiv 0$ in dimension $n\ge 5$. Interestingly enough, in dimension $n=4$ the function $U_{\ln {}}$ vanishes only when $d U \equiv 0$; in which case the space-time is vacuum, see Remark 5.3 below.

The proof of Theorem 1.1 can be found at the end of Sect. 5. It follows closely [8] and proceeds through an implicit function argument, with the key isomorphism properties of the associated linearised operator borrowed from [8, 15], and presented in Sect. 3.

As already noted, a constant ${\widehat{U}}$ implies vacuum, by uniqueness of solutions. Hence, the energy density of the Maxwell field behaves as $\rho ^{4}$ for small $\rho$ if not identically zero [cf. (6.14) below], which leads to finite-total-energy configurations in space-dimensions $n=3$ and 4, but infinite matter energy in higher dimensions.

## Definitions, notations and conventions

Our definitions and conventions are identical to those in [8, Section 2]. Here, we simply recall that the Lichnerowicz Laplacian acting on a symmetric two-tensor field is defined as [6, § 1.143]

$$\begin{aligned} \Delta _Lh_{ij}=-\nabla ^k\nabla _kh_{ij}+R_{ik}h^k{_j}+R_{jk}h^k{_i}-2R_{ikjl}h^{kl}. \end{aligned}$$

The operator $\Delta _L+2n$ arises naturally when linearising the equation

2.1 $$\begin{aligned} {\text {Ric}}({{\mathfrak {g}}})=-n {{\mathfrak {g}}}, \end{aligned}$$

where ${\text {Ric}}({{\mathfrak {g}}})$ is the Ricci curvature of ${{\mathfrak {g}}}$, at a solution. We will say that a metric is *non-degenerate* if $\Delta _L+2n$ has no $L^2$-kernel.

## Isomorphism theorems

In this section, we recall two isomorphism results from [8, Section 3] and prove an isomorphism theorem on scalar fields, as needed in the remainder of this work. The reader might also want to consult the introductory comments in [8, Section 3], which remain valid in the current context.

### An isomorphism on two-tensors

We will need [8, Corollary 3.2]:

#### Corollary 3.1

Let $(\mathbb {S}^1 \times M,V^2 \mathrm{d}\varphi ^2 + g)$ be an asymptotically hyperbolic non-degenerate Riemannian manifold with $\partial _\varphi V = 0= \partial _\varphi g$. Consider the map

$$\begin{aligned} (W,h)\mapsto (l(W,h),L(W,h)), \end{aligned}$$

where

3.1 $$\begin{aligned} l(W,h)= & {} V\displaystyle \big [ (\nabla ^*\nabla +2n+V^{-1}\nabla ^*\nabla V+V^{-2}|\mathrm{d}V|^2) W\nonumber \\&+V^{-1}\nabla _jV\nabla ^jW -V^{-1}\nabla ^jV\nabla ^kVh_{kj}+\langle {\text {Hess}}_gV,h\rangle _g \big ], \end{aligned}$$

and

3.2 $$\begin{aligned} L_{ij}(W,h)= & {} \frac{1}{2}\Delta _L h_{ij}+nh_{ij}-\frac{1}{2}V^{-1}\nabla ^kV\nabla _kh_{ij}\nonumber \\&+\frac{1}{2}V^{-2}(\nabla _iV\nabla ^kVh_{kj}+\nabla _jV\nabla ^kVh_{ki})\nonumber \\&-\frac{1}{2}V^{-1}(\nabla _i\nabla ^kVh_{kj}+\nabla _j\nabla ^kVh_{ki})\nonumber \\&+2V^{-2}W ({\text {Hess}}_g V)_{ij} -2V^{-3}\nabla _iV\nabla _jV W. \end{aligned}$$

Then (*l*, *L*) is an isomorphism from $C^{k+2,\alpha }_{\delta -1}(M)\times C^{k+2,\alpha }_{\delta }( M,{{\mathcal {S}}}_2)$ to $C^{k,\alpha }_{\delta -2}(M)\times C^{k,\alpha }_{\delta }( M,{\mathcal {S}}_2)$ when $\delta \in (0,n)$.

### An isomorphism on one-forms

We will also need the following sharper version of [8, Corollary 3.5], where we note that the isomorphism given in Theorem 3.4 and Corollary 3.5 of [8] are in fact valid for a larger interval of weights, as can be established by a more careful inspection of the arguments presented in [8]:

#### Corollary 3.2

Let $k\in {\mathbb {N}}$, $\alpha \in (0,1)$. Under the hypotheses of Corollary 3.1, suppose moreover that the Ricci tensor of $V^2 d\varphi ^2 + g$ is negative. Consider the operator

$$\begin{aligned} \Omega _i \mapsto {B}(\Omega )_i+R_{ij}\Omega ^j-V^{-1}\nabla _i\nabla ^jV\Omega _j=:{{\mathcal {B}}}(\Omega )_i, \end{aligned}$$

where

$$\begin{aligned} {B}(\Omega )_i:=\nabla ^k\nabla _k\Omega _i+V^{-1}\nabla ^kV\nabla _k\Omega _i -V^{-2}\nabla _iV\nabla ^kV\Omega _k. \end{aligned}$$

Then ${\mathcal {B}}$ is an isomorphism from $C^{k+2,\alpha }_{\delta }( M,{{\mathcal {T}}}_1)$ to $C^{k,\alpha }_{\delta }(M,{{\mathcal {T}}}_1)$ when

$$\begin{aligned} \left| \delta -\frac{n}{2}\right| <\frac{n+2}{2}. \end{aligned}$$

### An isomorphism on functions

If we assume that $V^2\mathrm{d}\varphi ^2+g$ is a static asymptotically hyperbolic metric on $\mathbb {S}^1\times M$, then it is easy to check that

3.3 $$\begin{aligned} V^{-2}|\mathrm{d}V|^2\rightarrow 1\text { and }V^{-1}\nabla ^i\nabla _iV\rightarrow n \end{aligned}$$

as the conformal boundary is approached. We will need an isomorphism property for the following operator acting on functions with $s=-1$; in [8] the result has already been established with $s=-3$ and $s=3$, so for future reference it appears useful to consider all values of *s*:

$$\begin{aligned} \sigma \mapsto {{\mathcal {T}}}_s\sigma :=V^{-s}\nabla ^i(V^{s}\nabla _i\sigma )= \nabla ^i\nabla _i\sigma +sV^{-1}\nabla ^iV\nabla _i\sigma . \end{aligned}$$

#### Theorem 3.3

Let (*M*, *g*) be an *n*-dimensional Riemannian manifold with an asymptotically hyperbolic metric with $V>0$ and assume that (3.3) holds. Let $s\ne 1-n$ and suppose that

$$\begin{aligned} \frac{s+n-1-|s+n-1|}{2}<\delta <\frac{s+n-1+|s+n-1|}{2}. \end{aligned}$$

If $s\ge -\frac{n-1}{2}$ then ${\mathcal {T}}_s$ is an isomorphism from $C^{k+2,\alpha }_{\delta }( M)$ to $C^{k,\alpha }_{\delta }(M)$. If $s<-\frac{n-1}{2}$, then ${{\mathcal {T}}}_s$ is an isomorphism from $C^{k+2,\alpha }_{\delta }( M)/\mathbb {R}$ to

3.4 $$\begin{aligned} \Big \{\sigma \in C^{k,\alpha }_{\delta }(M): \int _M V^{s}\sigma =0\Big \}. \end{aligned}$$

#### Proof

When $s+n-1>0$, we can use [5, Theorem 7.2.1 (ii) and Remark (i), p. 77] to conclude. As we want the result for any $s\ne 1-n$, for the remaining cases we appeal to the results of Lee [15]. For this, we need a formally self-adjoint operator, so we set $\sigma =V^{-\frac{s}{2}}f$, thus

3.5 $$\begin{aligned} {{\mathcal {T}}}_s\sigma =V^{{-}\frac{s}{2}}\left[ \nabla ^i\nabla _if-\frac{s}{2}\left( \left( \frac{s}{2}-1\right) V^{-2}|\mathrm{d}V|^2+V^{-1}\nabla ^i\nabla _iV\right) f\right] =: V^{{-}\frac{s}{2}}T_sf. \end{aligned}$$

By assumption $V^{-2}|\mathrm{d}V|^2\rightarrow 1$ and $V^{-1}\nabla ^i\nabla _iV\rightarrow n$ at the conformal boundary, leading to the following indicial exponents for $T_s$:

$$\begin{aligned} \delta =\frac{n-1\pm |s+n-1|}{2}. \end{aligned}$$

We want to show that $T_s$ satisfies condition (1.4) of [15],

3.6 $$\begin{aligned} \Vert u\Vert _{L^2}\le C \Vert { T}_su\Vert _{L^2}, \end{aligned}$$

for smooth *u* compactly supported in a sufficiently small open set ${{\mathcal {U}}}\subset M$ such that $\overline{{\mathcal {U}}}$ is a neighbourhood of $\partial M$. Let us recall the following, well-known result (see eg. [8, Lemma 3.8]):

#### Lemma 3.4

On an asympotically hyperbolic manifold (*M*, *g*) with boundary defining function $\rho$ we have, for all compactly supported $C^2$ functions,

$$\begin{aligned} \int u\nabla ^*\nabla u\ge \left( \frac{n-1}{2}\right) ^2\int (1+O(\rho )) u^2. \end{aligned}$$

Lemma 3.4 combined with the hypothesis (3.3) shows that

$$\begin{aligned} \Vert u\Vert _{L^2}\Vert T_su\Vert _{L^2}\ge -\int uT_su \ge \int \Big (\frac{(n-1)^2}{4}+\frac{s}{2}(\frac{s}{2}+n-1)+o(1) \Big )u^2, \end{aligned}$$

from which it follows that $$ T_s$$ satisfies indeed (3.6) with

$$\begin{aligned} C^{-1}=\frac{(s+n-1)^2}{4}. \end{aligned}$$

We recall that the critical fall-off for a function to belong to $L^2$ is $O(\rho ^{\frac{n-1}{2}})$. This can be used to show that the $L^2$-kernel of $T_s$ equals

$$\begin{aligned} \ker T_s=V^{\frac{s}{2}}\mathbb {R}\cap L^2= \left\{ \begin{array}{l}\{0\}\quad \text{ if } s\ge -\frac{n-1}{2},\\ V^{\frac{s}{2}}\mathbb {R}\quad \text{ if } s<-\frac{n-1}{2}. \end{array}\right. \end{aligned}$$

Indeed, assume that *f* is in the $L^2$-kernel of $T_s$. By elliptic regularity (see [15, Lemma 4.8] for instance) *f* is $H^2$ on *M*. This implies in particular that no boundary terms arise in the following integration by parts:

$$\begin{aligned} 0= & {} - \int fT_s f =-\int f V^{-\frac{s}{2}} \nabla ^i[V^{s}\nabla _i(V^{-\frac{s}{2}}f)]\\= & {} \int V^{s}|\nabla (V^{-\frac{s}{2}}f)|^2. \end{aligned}$$

So $V^{-\frac{s}{2}}f$ is a constant. Using [15], Theorem C(c), we find:

- If $s\ge \frac{n-1}{2}$, then the kernel (and then the cokernel) of $T_s$ is trivial so $T_s$ is an isomorphism from $C^{k+2,\alpha }_{\delta -\frac{s}{2}}( M)$ to $C^{k,\alpha }_{\delta -\frac{s}{2}}( M)$.
- If $s<-\frac{n-1}{2}$, then $T_s$ is an isomorphism from $C^{k+2,\alpha }_{\delta -\frac{s}{2}}( M)/V^{s/2}\mathbb {R}$ to the orthogonal to the kernel:
  $$\begin{aligned} \Big \{f\in C^{k,\alpha }_{\delta -\frac{s}{2}}(M): \int _M V^{s/2}f=0\Big \}. \end{aligned}$$

The conclusion follows for ${\mathcal {T}}_s$ if we recall that $\sigma =V^{-\frac{s}{2}}f$ is in $C^{k,\alpha }_{\delta }(M)$ iff $f\in C^{k,\alpha }_{\delta -\frac{s}{2}}(M)$. $\square$

## The equations

Rescaling the metric to achieve a convenient normalisation of the cosmological constant,

4.1 $$\begin{aligned} \Lambda =-\frac{n(n-1)}{2}, \end{aligned}$$

the vacuum Einstein–Maxwell equations for a metric satisfying (1.3)–(1.4) read (see “Appendix”)

4.2 $$\begin{aligned} \left\{ \begin{array}{l} V(\nabla ^*\nabla V+n V)=-\frac{n-2}{n-1} |\mathrm{d}U|_g^2,\\ {\text {Ric}}(g)+n g-V^{-1}{\text {Hess}}_gV=V^{-2}\left( -\mathrm{d}U\otimes \mathrm{d}U+\frac{1}{n-1}|\mathrm{d}U|_g^2g\right) ,\\ {\text {div}}_g (V^{-1} \nabla U)=0. \end{array}\right. \end{aligned}$$

### The linearised equation

We use the symbol ${\text {Tr}}$, or ${\text {Tr}}_g$ when the metric could be ambiguous, to denote the trace. As in [8] we set

$$\begin{aligned} {\text {grav}}h=h-\frac{1}{2}{\text {Tr}}_g hg,\,\,\, ({\text {div}}h)_i=-\nabla ^kh_{ik},\,\,\, ({\text {div}}^*w)_{ij}=\frac{1}{2}(\nabla _iw_j+\nabla _jw_i) \end{aligned}$$

(note the geometers’ convention, in which we have a negative sign in the definition of divergence). We consider the operator from the set of functions times symmetric two-tensor fields to itself, defined as

$$\begin{aligned} \left( \begin{array}{l}V\\ g\end{array}\right) \mapsto \left( \begin{array}{l}V(\nabla ^*\nabla V+nV)\\ {\text {Ric}}(g)+ng-V^{-1}{\text {Hess}}_g V\end{array}\right) . \end{aligned}$$

(To avoid ambiguities, $\nabla ^*\nabla \equiv -\nabla ^i \nabla _i =: - \Delta$.) The two components of its linearisation at (*V*, *g*) are

$$\begin{aligned} p(W,h)= & {} V\Big [(\nabla ^*\nabla +2n+V^{-1}\nabla ^*\nabla V) W + \langle {\text {Hess}}_gV,h\rangle _g\\&-\langle {\text {div}}{\text {grav}}h,dV\rangle _g\Big ], \\ P_{ij}(W,h)= & {} \frac{1}{2}\Delta _Lh_{ij}+nh_{ij}+\frac{1}{2}V^{-1}\nabla ^kV(\nabla _ih_{kj}+ \nabla _jh_{kj}-\nabla _kh_{ij})\\&-({\text {div}}^* {\text {div}}{\text {grav}}h)_{ij}+V^{-2}W({\text {Hess}}_gV)_{ij}-V^{-1}({\text {Hess}}_g W)_{ij}. \end{aligned}$$

It turns out to be convenient to introduce the one-form

$$\begin{aligned} w_j=V^{-1}\nabla ^kVh_{kj}+\nabla ^kh_{kj}-\frac{1}{2}\nabla _j({\text {Tr}}h)-V^{-1}\nabla _jW-V^{-2}\nabla _jV W, \end{aligned}$$

which allows us to rewrite *P*(*W*, *h*) as

$$\begin{aligned} P(W,h)= & {} L(W,h)+{\text {div}}^*w, \end{aligned}$$

where *L* is as in Corollary 3.1. Similarly, *p*(*W*, *h*) can be rewritten as

$$\begin{aligned} p(W,h)= & {} l(W,h)+V\langle w,\mathrm{d}V\rangle _g, \end{aligned}$$

with *l* given by (3.1).

### The modified equation

In Sect. 5, we will use the implicit function theorem to construct our solutions, using the observation of [11], how to obtain a well-behaved equation by adding “gauge fixing terms”. We choose those terms as in [8], appealing to harmonic maps for the vacuum Einstein equations in one dimension higher.

Indeed, we will be solving the following system of equations

4.3 $$\begin{aligned} \left\{ \begin{array}{l} V(\nabla ^*\nabla V+n V +\langle \Omega ,\mathrm{d}V\rangle )+\frac{n-2}{n-1} |\mathrm{d}U|_g^2=0,\\ {\text {Ric}}(g)+n g-V^{-1}{\text {Hess}}_gV+{\text {div}}^*\Omega \\ \qquad -V^{-2}\left( -\mathrm{d}U\otimes \mathrm{d}U+\frac{1}{n-1}|\mathrm{d}U|_g^2g\right) =0,\\ {\text {div}}_g (V^{-1} \nabla U)=0, \end{array}\right. \end{aligned}$$

where

4.4 $$\begin{aligned} -\Omega _j\equiv & {} -\Omega (V,g, {{\mathring{V}}}, {{\mathring{g}}})_j\nonumber \\ \nonumber:= & {} {{\mathfrak {g}}}_{j\mu }{{\mathfrak {g}}}^{\alpha \beta } (\Gamma ({{\mathfrak {g}}})^\mu _{\alpha \beta }- \Gamma (\mathring{{{\mathfrak {g}}}})^\mu _{\alpha \beta })\nonumber \\= & {} {g}{}^{\ell m} (\mathring{\nabla }_m {g}_{j\ell }- \frac{1}{2} \mathring{\nabla }_jg_{\ell m}) + V^{-2}g_{jk}( {{\mathring{V}}}\mathring{\nabla }^k {{\mathring{V}}}- V\nabla ^k V), \end{aligned}$$

and where ${\mathring{\nabla }}$-derivatives are relative to the metric ${\mathring{g}}$, with Christoffel symbols $\mathring{\Gamma }^\alpha _{\beta \gamma }$, latin indices run from 0 to *n*, and ${{\mathfrak {g}}}:=V^2(dx^0)^2+g$ with Christoffel symbols $\Gamma ({{\mathfrak {g}}})^\alpha _{\beta \gamma }$, while the $\Gamma (\mathring{{{\mathfrak {g}}}})^\alpha _{\beta \gamma }$’s are the Christoffel symbols of the metric $\mathring{{{\mathfrak {g}}}}$.

The derivative of $\Omega$ with respect to (*V*, *g*) at $( {{\mathring{V}}}, {{\mathring{g}}})$ is

$$\begin{aligned} D_{(V,g)}\Omega ( {{\mathring{V}}}, {{\mathring{g}}}, {{\mathring{V}}}, {{\mathring{g}}})(W,h)=-w, \end{aligned}$$

where *w* is the one-form defined in Sect. 4.1 with (*V*, *g*) replaced with $( {{\mathring{V}}}, {{\mathring{g}}})$. Thus, the linearisation of (*q*, *Q*) at $( {{\mathring{V}}}, {{\mathring{g}}})$ is

$$\begin{aligned} D(q,Q)( {{\mathring{V}}}, {{\mathring{g}}})=(l,L), \end{aligned}$$

where (*l*, *L*) is the operator defined in Sect. 4.1 with (*V*, *g*) replaced with $( {{\mathring{V}}}, {{\mathring{g}}})$. We will show that, under reasonable conditions, solutions of (4.3) are solutions of (4.2).

#### Proposition 4.1

If (*U*, *V*, *g*) solves (4.3) then $\Omega$ is in the kernel of ${{\mathcal {B}}}$. If moreover $\Omega \in C^{2,\alpha }_\delta$ for some $\delta >-1$, then $\Omega \equiv 0$, so that (*U*, *V*, *g*) solves (4.2).

#### Proof

A standard adaptation of the argument in [8] gives the result; we sketch this for completeness. Set

$$\begin{aligned} a:= & {} -\frac{n-2}{n-1} V^{-2} |\mathrm{d} U|^2,\\ A:= & {} V^{-2}\left( -\mathrm{d}U\otimes \mathrm{d}U+\frac{1}{n-1} |\mathrm{d} U|^2 g\right) . \end{aligned}$$

With this notation, the first two equations in (4.3) take the form

4.5 $$\begin{aligned} \left\{ \begin{array}{l} \nabla ^*\nabla V+n V+\langle \Omega ,\mathrm{d}V\rangle =V a,\\ {\text {Ric}}(g)+n g-V^{-1}{\text {Hess}}_gV+{\text {div}}^*\Omega = A, \end{array}\right. \end{aligned}$$

The tensor field, $E(g)={\text {grav}}_g {\text {Ric}}(g)$, has vanishing divergence, which provides the equation ${{\mathcal {B}}}(\Omega )=0$ for $\Omega$. Indeed, the operator ${{\mathcal {B}}}(\Omega )$ defined above has been constructed in [8] so that it vanishes when the modified equation for the metric holds and when the Lorentzian $(n+1)$-dimensional divergence of the $(n+1)$-dimensional energy–momentum tensor vanishes. The latter condition is satisfied when the $(n+1)$-dimensional matter field equations hold; in the current case, this coincides with the last equation in (4.3).

An alternative, *n*-dimensional argument proceeds as follows: the calculation following [8, Equation (4.6)] shows that for a solution to the modified equation, the divergence of *E*(*g*) equals

$$\begin{aligned} 0\equiv {\text {div}}E(g)=\frac{1}{2}{{\mathcal {B}}}(\Omega ) + \beta , \end{aligned}$$

where

4.6 $$\begin{aligned} \beta :=V^{-1}\mathrm{d}(Va) - V^{-1} A(\nabla V,\cdot )+ {\text {div}}\left( {\text {grav}}_g A+\frac{a}{2}g\right) . \end{aligned}$$

The vanishing of $\beta$ can be checked by a somewhat lengthy calculation using (4.3).

Either way, the Bianchi identity ${\text {div}}E(g)=0$ shows that $\Omega$ is in the kernel of ${{\mathcal {B}}}$. It follows from Corollary 3.2 that the only solution of this equation which decays as described is zero.$\square$

For future reference, we consider the static equations, modified as in (4.5), with a general energy–momentum tensor:

4.7 $$\begin{aligned}&-V^ {-1} \nabla ^i \nabla _i V - \frac{ 2 \Lambda }{n-1} + V^{-1} \nabla ^i\Omega \nabla _i V= \underbrace{ - T_{\alpha \beta } N^\alpha N^\beta - \frac{g^{\alpha \beta }T_{\alpha \beta } }{n-1}} _{ =: a} , \end{aligned}$$

4.8 $$\begin{aligned}&R_{ij}- V^{-1} \nabla _i\nabla _j V - \frac{2\Lambda }{n-1} g_{ij} +\frac{1}{2} (\nabla _i \Omega _j + \nabla _j \Omega _i)= \underbrace{ T_{ij} - \frac{g^{\alpha \beta }T_{\alpha \beta } }{n-1} g_{ij}}_{=:A_{ij}} , \qquad \end{aligned}$$

where $N^\alpha \partial _\alpha$ is the unit timelike normal to the initial data surface [compare (6.8)–(6.9), Appendix]. We have:

#### Proposition 4.2

Let $T_{\mu \nu }$ be divergence-free with respect to the time-independent metric

$$\begin{aligned} {{\mathbf {g}}}= -V^2 \mathrm{d}t^2 + g \end{aligned}$$

as a consequence of the matter field equations, with $\partial _t T_{\mu \nu } = T_{0i}=0$. Set

4.9 $$\begin{aligned} a:= - T_{\alpha \beta } N^\alpha N^\beta - \frac{g^{\alpha \beta }T_{\alpha \beta } }{n-1} , \quad A_{ij} := T_{ij} - \frac{g^{\alpha \beta }T_{\alpha \beta } }{n-1} g_{ij}. \end{aligned}$$

Then

4.10 $$\begin{aligned} \beta \equiv 0. \end{aligned}$$

#### Proof

Equation (4.6) can be rewritten as

4.11 $$\begin{aligned} -\beta _j= & {} \nabla _i\big ( A^i{}_j - \frac{1}{2} (A^k{}_k + {a}) \delta ^i_j\big ) + V^{-1} \big (A_{ij} - a g_{ij})\nabla V ^i\nonumber \\= & {} \nabla _i\Big ( {T^i{}_j - \frac{1}{2} (\frac{2g^{\alpha \beta }T_{\alpha \beta } }{n-1} +A^k{}_k + {a}) \delta ^i_j}\Big )\nonumber \\&+ V^{-1} \left( T_{ij} - \left( \frac{ g^{\alpha \beta }T_{\alpha \beta } }{n-1} - T_{\alpha \beta } N^\alpha N^\beta - \frac{g^{\alpha \beta }T_{\alpha \beta } }{n-1}\right) g_{ij}\right) \nabla V ^i . \qquad \end{aligned}$$

We have

$$\begin{aligned} \frac{2g^{\alpha \beta }T_{\alpha \beta } }{n-1} + A^k{}_k +a= & {} \frac{2g^{\alpha \beta }T_{\alpha \beta } }{n-1} + g^{ij} T_{ij} - \frac{n }{n-1}g^{\alpha \beta }T_{\alpha \beta } \\&- T_{\alpha \beta } N^\alpha N^\beta - \frac{g^{\alpha \beta }T_{\alpha \beta } }{n-1} =0 . \end{aligned}$$

The $(n+1)$-decomposition of the equation $\nabla ^\mu T_{\mu \nu }=0$, where $\nabla _\mu$ is the space-time covariant derivative associated with ${{\mathbf {g}}}$, using the formulae for the Christoffel symbols in “Appendix” gives

4.12 $$\begin{aligned} \nabla ^i T_{ij} +V T_{\alpha \beta } N ^ \alpha N^\beta \nabla _j V + V^{-1} \nabla ^j V T_{ij} = 0, \end{aligned}$$

where $\nabla ^i$ is the covariant derivative operator of *g*. Comparing with (4.11) gives the result. $\square$

## The construction

We consider an asymptotically hyperbolic Einstein static metric $\mathring{{{\mathfrak {g}}}}={{\mathring{V}}}^2\mathrm{d}\varphi ^2+{{\mathring{g}}}$ on $\mathbb {S}^1\times M$. We apply Theorem 3.3 with $s=-1$, *g*—a Riemannian metric on *M* close to ${{\mathring{g}}}$ in $C_{0}^{k+2,\alpha }(M,{{\mathcal {S}}}_2)$, and *V*—a function on *M* close to ${{\mathring{V}}}$ in $C_{-1}^{k+2,\alpha }(M)$. It is convenient to choose some

5.1 $$\begin{aligned} \delta \in (0,1)\text { when }n=3\text { and }\delta =1\text { if }n>3. \end{aligned}$$

We conclude that for any $\widehat{U}\in C^{k+2,\alpha }(\partial M)$, there exists a unique solution

$$\begin{aligned} U=U(\widehat{U},V,g)\in C_{0}^{k+2,\alpha }(M) \end{aligned}$$

to

$$\begin{aligned} \left\{ \begin{array}{l} \nabla ^*(V^{-1}\nabla U)=0,\\ U-\widehat{U}\in C_{\delta }^{k+2,\alpha }(M). \end{array}\right. \end{aligned}$$

Moreover, the map $(\widehat{U},V,g)\mapsto U-\widehat{U}\in C_{\delta }^{k+2,\alpha }(M)$ is smooth.

We define a new map *F*, defined on the set of functions on the conformal boundary at infinity $\partial _\infty M$ times functions on *M* times symmetric two-tensor fields on *M*, mapping to functions on *M* times symmetric two-tensor fields on *M*, which to $(\widehat{U},V,g)$ associates

$$\begin{aligned} \left( \begin{array}{c}V(\nabla ^*\nabla V+nV+\langle \Omega (V,g,{{\mathring{V}}},{{\mathring{g}}}),\mathrm{d}V\rangle )+\frac{n-2}{n-1} |\mathrm{d} U|^2\\ {\text {Ric}}(g)+ng-V^{-1}\nabla _i\nabla _jV+{\text {div}}^*\Omega (V,g,{{\mathring{V}}},{{\mathring{g}}})+ V^{-2}(\mathrm{d} U\otimes \mathrm{d} U-\frac{1}{n-1}|\mathrm{d} U|^2 g)\end{array}\right) . \end{aligned}$$

### Proposition 5.1

Let $\mathring{{{\mathfrak {g}}}}={{\mathring{V}}}^2d\varphi ^2+{{\mathring{g}}}$ be an asymptotically hyperbolic static Einstein metric on $\mathbb {S}^1\times M$, $k\in {\mathbb {N}}$, $\alpha \in (0,1)$. The map ${\mathcal {F}}$ defined as

$$\begin{aligned}&C^{k+2,\alpha }(\partial M)\times C_{1}^{k+2,\alpha }(M)\times C_{2}^{k+2,\alpha }(M,{{\mathcal {S}}}_2)\longrightarrow C_{0}^{k,\alpha }(M)\times C_{ {2} }^{k,\alpha }(M,{\mathcal {S}}_2)\\&(\widehat{U},W,h) \longmapsto F(\widehat{U},{{\mathring{V}}}+W,{{\mathring{g}}}+h) \end{aligned}$$

is smooth in a neighbourhood of zero.

### Proof

Let $\delta$ be as in (5.1). We have, by direct estimations,

$$\begin{aligned} V^{-2} \left( \mathrm{d}U\otimes \mathrm{d}U-\frac{1}{n-1} |\mathrm{d} U|^2 g\right) \in {C_{2 + 2 \delta }^{k,\alpha }(M,{{\mathcal {S}}}_2)\subset } C_{2}^{k,\alpha }(M,{{\mathcal {S}}}_2). \end{aligned}$$

The remaining arguments of the proof of [8, Proposition 5.2] apply with trivial modifications: the function ${{\mathring{V}}}\in C_{-1}^{k+2,\alpha }(M)$ is strictly positive, so the same is true for ${{\mathring{V}}}+W$ if *W* is sufficiently small in $C_{1}^{k+2,\alpha }(M)\subset C_{-1}^{k+2,\alpha }(M)$. Similarly, the symmetric two-tensor field ${{\mathring{g}}}+h\in C_{0}^{k+2,\alpha }(M,{{\mathcal {S}}}_2)$ is positive definite when *h* is small in $C_{2}^{k+2,\alpha }(M,{{\mathcal {S}}}_2)\subset C_{0}^{k+2,\alpha }(M,{{\mathcal {S}}}_2)$. The map $(\widehat{U},V,g)\mapsto U$ is smooth. The fact that the remaining terms in $F(\widehat{U},{{\mathring{V}}}+W,{{\mathring{g}}}+h)$ are in the space claimed and that the map is smooth, follows from standard calculations (see [12, proof of Theorem 4.1] or [10, Appendix] for associated detailed calculations). $\square$

We are ready to formulate now:

### Theorem 5.2

Let $\dim M=n\ge 3$ and let ${{\mathring{V}}}^2d\varphi ^2+{{\mathring{g}}}$ be a non-degenerate asymptotically hyperbolic static Einstein metric on $\mathbb {S}^1\times M$, $k\in {\mathbb {N}}$, $\alpha \in (0,1)$, $\delta \in (0,1)$ in $n=3$ and $\delta =1$ if $n>3$. For all $\widehat{U}$ close to zero in $C^{k+2,\alpha }(\partial M)$ there exists a unique solution

$$\begin{aligned} (U,V,g)=(\widehat{U}+u,{{\mathring{V}}}+W,{{\mathring{g}}}+h) \end{aligned}$$

to (4.2) with

$$\begin{aligned} (u,W,h)\in C_{\delta }^{k+2,\alpha }(M)\times C_{1}^{k+2,\alpha }(M)\times C_{2}^{k+2,\alpha }(M,{{\mathcal {S}}}_2), \end{aligned}$$

close to zero, satisfying the gauge condition $\Omega =0$. Moreover, the map $\widehat{U}\mapsto (u,W,h)$ is a smooth map of Banach spaces near zero.

### Proof

As already pointed out, the function $U=U(\widehat{U},V,g)$ exists and is unique when *W* and *h* are small. From Proposition 5.1, we know that the map ${{\mathcal {F}}}$ is smooth. The linearisation of ${{\mathcal {F}}}$ at zero is

$$\begin{aligned} D_{(W,h)}{\mathcal {F}}(0,0,0)=D_{(V,g)}F(0,{{\mathring{V}}},{{\mathring{g}}})=(l,L). \end{aligned}$$

From Corollary 3.1, with $\delta =2$, we obtain that $D_{(W,h)}{{\mathcal {F}}}(0,0,0)$ is an isomorphism. The implicit function theorem shows that the conclusion of Theorem 5.2 remains valid *for the modified equation* (4.3). Returning to Sect. 4.2, we see that $\Omega =\Omega (V,g,{{\mathring{V}}},{{\mathring{g}}})\in C_{2}^{k+1,\alpha }(M,T_1)$. Corollary 3.2 gives $\Omega =0$, and we have obtained a solution to (4.2).$\square$

### Proof of Theorem 1.1

Existence and uniqueness of a solution with

5.2 $$\begin{aligned} V-{\mathring{V}}=O(\rho ),\quad U={\widehat{U}} +O(\rho ^{\delta }) ,\quad g-{\mathring{g}} ={O_{\mathring{g}}}(\rho ^2)={O_{\rho ^2 \mathring{g}}}(1), \end{aligned}$$

follows from Theorem 5.2. Here and elsewhere, we write $u=O(\rho ^\sigma )$ for a tensor *u* if the coordinate components of *u* in local coordinates near the boundary are $O(\rho ^\sigma )$, and $u={O_{\mathring{g}}}(\rho ^\sigma )$ if the norm $|u|_{{\mathring{g}}}$ of *u* with respect to the metric $\mathring{g}$ is $O(\rho ^\sigma )$. Standard arguments show that all the fields *V*, *U* and *g* are polyhomogeneous (cf. e.g. [9], compare [8, Section 7]).

To justify (1.7), we plug-in a polyhomogeneous asymptotics as in (5.2) in the equations. Since the trace-free part of the Ricci tensor of ${{\mathbf {g}}}=-V^2\mathrm{d}t^2+g$ decays in $\mathring{{{\mathfrak {g}}}}$-norm as $\rho ^4$ near $\rho =0$, the metric ${{\mathbf {g}}}=-V^2\mathrm{d}t^2+g$ is vacuum up to this order, which implies that the usual Fefferman–Graham expansion holds for ${{\mathbf {g}}}$ up to terms $O_{\mathring{{{\mathfrak {g}}}}}(\rho ^4 \ln \rho )$. This leads to the following form of the metric near the conformal boundary

5.3 $$\begin{aligned} {\mathring{g}}=\rho ^{-2}(\mathrm{d}\rho ^2+{\mathring{h}}),\quad {\mathring{h}}=\breve{h}+O_{\breve{h}}(\rho ^2),\quad {\mathring{V}}=\breve{V}\rho ^{-1}+O(\rho ) , \end{aligned}$$

where ${\mathring{h}}$ is a family of Riemannian metrics on the boundary depending upon $\rho$, with

$$\begin{aligned} g={\mathring{g}}+ O_{{\mathring{g}}}(\rho ^2), \quad V={\mathring{V}}+O(\rho ). \end{aligned}$$

The equation $\nabla _i(V^{-1}\nabla ^i U)=0$ in local coordinates $(\rho ,x^A)$ as in (5.3) takes the form

$$\begin{aligned}&\breve{V}^{-1}\sqrt{\det \breve{h}}\;\partial _\rho \left[ \rho ^{3-n}(1+O(\rho ^2))\partial _\rho U\right] \\&\quad =-\rho ^{3-n}\partial _A\left[ \left( \breve{V}^{-1}\sqrt{\det \breve{h}}+O(\rho ^2)\right) (\breve{h}^{AB}+O^{AB}(\rho ^2))\partial _BU\right] \\&\qquad +\partial _\rho \left( \rho ^{5-n}O^A(1)\partial _A U\right) +\partial _A\left( \rho ^{5-n}O^A(1)\partial _\rho U\right) . \end{aligned}$$

Let *U* be a polyhomogenous solution with $U={\widehat{U}}+O(\rho ^\delta )$. When $n=3$, we can see that no logarithmic term $O(\rho \ln \rho )$ is needed in *U* so that $U={\widehat{U}}+O(\rho )$. When $n=4$, we find

5.4 $$\begin{aligned} U={\widehat{U}} \underbrace{ {-} \frac{1}{2}\breve{V}\breve{\nabla }_A(\breve{V}^{-1}\breve{\nabla }^A{\widehat{U}})}_{=: U_{\ln {}}}\rho ^2\ln \rho +O(\rho ^2). \end{aligned}$$

(In dimensions $n\ge 5$ on expects in general a logarithmic term $O(\rho ^{n-2} \ln \rho )$ in an asymptotic expansion of *U*.) $\square$

### Remark 5.3

The equality

$$\begin{aligned} 2\int _{\partial M}\breve{V}^{-1}{\widehat{U}}U_{\ln {}}=\int _{\partial M}\breve{V}^{-1}|\breve{\nabla }{\widehat{U}}|^2, \end{aligned}$$

together with the definition of $U_{\ln }$, proves that $U_{\ln {}}=0$ if and only if ${{\widehat{U}}}$ is constant. When ${\widehat{U}}$ is constant the function $U:= {\widehat{U}}$ solves the equations and satisfies the right-boundary values, so the associated solution is vacuum.

## Appendix: Static Einstein–Maxwell equations in dimensions $n+1\ge 4$

Consider a Riemannian manifold (*M*, *g*), let us denote by $\nabla$ the Levi-Civita derivative operator associated with *g*. Let $V:M\rightarrow \mathbb {R}$, and set

$$\begin{aligned} ({\mathscr {M}}=\mathbb {R}\times M, \, \widetilde{ g}=\varepsilon V^2\mathrm{d}t^2+g),\quad \varepsilon =\pm 1, \end{aligned}$$

and $(x^\mu )=(x^0=t,x^i=(x^1,\ldots ,x^n))$. We have

6.1 $$\begin{aligned}&\widetilde{\Gamma }^0_{00}=\widetilde{\Gamma }^0_{ij}=\widetilde{\Gamma }^k_{i0}=0,\quad \widetilde{\Gamma }^0_{i0}=V^{-1}\partial _iV, \quad \widetilde{\Gamma }^k_{00}=-\varepsilon V\nabla ^kV, \quad \widetilde{\Gamma }^k_{ij}={\Gamma }^k_{ij},\nonumber \\ \end{aligned}$$

6.2 $$\begin{aligned}&\widetilde{R}^l{}_{ijk}={R}^l{}_{ijk}, \quad \widetilde{R}^l{}_{0j0}=-\varepsilon V\nabla _j\nabla ^l V, \quad \widetilde{R}^0{}_{ij0}=V^{-1}\nabla _j\nabla _iV, \end{aligned}$$

6.3 $$\begin{aligned}&\widetilde{R}^0{}_{ijk}=\widetilde{R}^l{}_{ij0}= \widetilde{R}^l{}_{0jk}=0, \end{aligned}$$

6.4 $$\begin{aligned}&\widetilde{R}_{ik}={R}_{ik}-V^{-1}\nabla _k\nabla _iV,\quad \widetilde{R}_{0k}=0,\quad \widetilde{R}_{00}=-\varepsilon V\nabla ^i\nabla _iV, \end{aligned}$$

6.5 $$\begin{aligned}&\widetilde{R}= R-2V^{-1}\nabla ^i\nabla _iV. \end{aligned}$$

Consider the $(n+1)$-dimensional Einstein equations

6.6 $$\begin{aligned} {\tilde{R}}_{\alpha \beta } -\frac{1}{2} {\tilde{R}} {\tilde{g}}_{\alpha \beta } +\Lambda {\tilde{g}}_{\alpha \beta } = T_{\alpha \beta } . \end{aligned}$$

Equations (6.6) with ${\tilde{g}} = -V^2 \mathrm{d}t^2 +g$ lead to

6.7 $$\begin{aligned}&\displaystyle {\widetilde{R}}_{\alpha \beta } = \frac{2\Lambda - {\text {Tr}}_{{\tilde{g}}} T}{n-1} {\widetilde{g}}_{\alpha \beta } + T_{\alpha \beta }, \end{aligned}$$

6.8 $$\begin{aligned}&\displaystyle R_{ij} = V^{-1} \nabla _i\nabla _j V + \frac{2\Lambda }{n-1} g_{ij} + T_{ij} - \frac{{\text {Tr}}_{{\tilde{g}}} T }{n-1} g_{ij} , \end{aligned}$$

6.9 $$\begin{aligned}&\displaystyle V \nabla ^i \nabla _i V = V^2\left( T_{\alpha \beta } N^\alpha N^\beta + \frac{{\text {Tr}}_{{\tilde{g}}} T - 2 \Lambda }{n-1} \right) , \end{aligned}$$

where $N^\alpha \partial _\alpha$ is the unit timelike normal to the level sets of *t*.

For a static electric field $F = \mathrm{d} (U \mathrm{d}t)$, $\partial _t U =0$, and $T_{\mu \nu }$ as in (1.1) we find

6.10 $$\begin{aligned}&|F|^2 = - 2 V^{-2} |\mathrm{d}U|^2, \end{aligned}$$

6.11 $$\begin{aligned}&{{\tilde{g}}^{\mu \nu } T_{\mu \nu }} \equiv {\text {Tr}}_{{\tilde{g}}} T= \frac{(3-n)}{4} |F|^2 = - \frac{(3-n)}{2} V^{-2}|\mathrm{d}U|^2 , \end{aligned}$$

6.12 $$\begin{aligned}&T_{ij} = -V^{-2} \nabla _i U \nabla _j U +\frac{1}{2} V^{-2}|\mathrm{d}U|^2 g_{ij} , \end{aligned}$$

6.13 $$\begin{aligned}&g^{ij} T_{ij} = \frac{ (n-2)}{2} V^{-2} |\mathrm{d}U|^2 \end{aligned}$$

6.14 $$\begin{aligned}&T_{{\widehat{0}} {\widehat{0}}} := T_{\mu \nu }N^\mu N^\nu = \frac{1}{2} V^{-2}|\mathrm{d}U|^2 . \end{aligned}$$

Choosing $\Lambda$ as in (4.1), Eq. (6.9) gives

6.15 $$\begin{aligned} V \Delta V= & {} \frac{n-2}{ (n-1)} |\mathrm{d}U|^2 -\frac{2 \Lambda V^2}{n-1} = \frac{n-2}{ (n-1)} |\mathrm{d}U|^2 + {n V^2} . \end{aligned}$$

When $\widetilde{ g}=\varepsilon V^2\mathrm{d}t^2+g$ is static and

6.16 $$\begin{aligned} \displaystyle T=T_{00}(\mathrm{d}x^0)^2+T_{ij}\mathrm{d}x^i\mathrm{d}x^j, \quad \partial _0T_{00}=\partial _0T_{ij}=0, \end{aligned}$$

then

6.17 $$\begin{aligned} \displaystyle {\tilde{\nabla }}^{\alpha }T_{\alpha \beta }\mathrm{d}x^\beta = \big (\nabla ^iT_{ij}+ V^{-1}\nabla ^iVT_{ij}-\varepsilon V^{-3}\nabla _jVT_{00}\big )\mathrm{d}x^j . \end{aligned}$$
